# Correction of Posterior Crossbite Using Rapid Maxillary Expansion in a Nine-Year-Old Boy: A Case Report

**DOI:** 10.7759/cureus.88078

**Published:** 2025-07-16

**Authors:** Tejal L Beldar, Ashwin M Jawdekar, Laresh N Mistry

**Affiliations:** 1 Department of Pediatric and Preventive Dentistry, Bharati Vidyapeeth (Deemed to be University) Dental College and Hospital, Navi Mumbai, IND

**Keywords:** crossbite, interceptive orthodontics, malocclusion, maxillary expansion, rapid palatal expansion

## Abstract

Maxillary constriction with a Class III malocclusion in the mixed dentition stage presents a unique challenge that requires early and customized orthodontic intervention. This case report highlights the management of a nine-year-old male patient with maxillary deficiency, an edge-to-edge incisor relationship, and a unilateral posterior crossbite using a modified Hyrax-type rapid maxillary expander (RME) with both anterior and posterior expansion capabilities. A fixed, bonded acrylic Hyrax-type RME with anterior extensions was designed to achieve three-dimensional maxillary expansion. Following appliance activation and subsequent breakage of the anterior segment, the posterior component was retained until the desired expansion was completed. A palatal crib appliance was delivered post-expansion to control the tongue thrust habit and stabilize the anterior open bite correction. Cephalometric analysis post-treatment demonstrated favorable skeletal and dentoalveolar changes, including increased SNA (79°-85°), reduced FMA (28°-22°), and improved Jarabak’s ratio (66.0-71.28), indicating horizontal growth and effective protraction of the maxilla. Dental changes included increased incisor proclination and improved overjet and occlusion. The interdisciplinary approach resulted in the correction of the crossbite, an improved facial profile, and normalization of oral function.

This case emphasizes the significance of early diagnosis and individualized appliance design in managing maxillary constriction and Class III tendencies. The combination of orthopedic expansion and habit correction successfully addressed both skeletal and functional components, demonstrating the potential for long-term occlusal stability and harmonious facial growth.

## Introduction

Posterior crossbite is one of the most frequently encountered transverse discrepancies in pediatric dentistry, with a reported prevalence ranging from 8% to 23% in the primary and mixed dentition stages [1,2]. The prevalence of crossbite varies widely across populations due to differences in genetics, environmental factors, age groups, and diagnostic criteria, with the global prevalence of posterior crossbite being approximately 7% to 23% of the population [3].

Posterior crossbite is defined as an abnormal buccolingual relationship between opposing molars or premolars, typically resulting from maxillary constriction, mandibular overgrowth, or a combination of both [4]. If left uncorrected, posterior crossbite can lead to functional mandibular shifts, facial asymmetry, abnormal occlusal wear, and temporomandibular joint dysfunction [5,6].

Early identification and timely intervention are crucial in managing posterior crossbite to prevent long-term skeletal and occlusal complications. Rapid maxillary expansion is a well-established orthopedic approach to correcting maxillary transverse deficiencies in growing children [7]. The technique involves applying controlled lateral forces to separate the midpalatal suture, allowing skeletal expansion of the maxilla and creating additional space in the dental arch [8,9]. The rapid maxillary expander (RME) not only addresses the underlying skeletal discrepancy but also improves nasal airflow and overall arch coordination [10].

This case report illustrates the successful correction of a unilateral posterior crossbite in a nine-year-old growing male using an RME. The report emphasizes clinical decision-making, treatment progression, and the benefits of interceptive orthodontics in pediatric patients. This case report is made as per the Case Report (CARE) checklist.

## Case presentation

A nine-year-old boy reported to the Department of Pediatric Dentistry of a private dental college with a chief complaint of malaligned teeth. Extraoral clinical examination showed the mesofacial facial type, with reduced vertical lower facial height. The chin appeared retruded, contributing to the convexity of the profile. The nasolabial angle was slightly acute, indicating a degree of upper lip protrusion or maxillary prominence. The lips were competent at rest, with no visible strain during closure. The soft tissue chin was mildly deficient, and the mentolabial sulcus appeared moderately deep (Figure 1).

**Figure 1:**
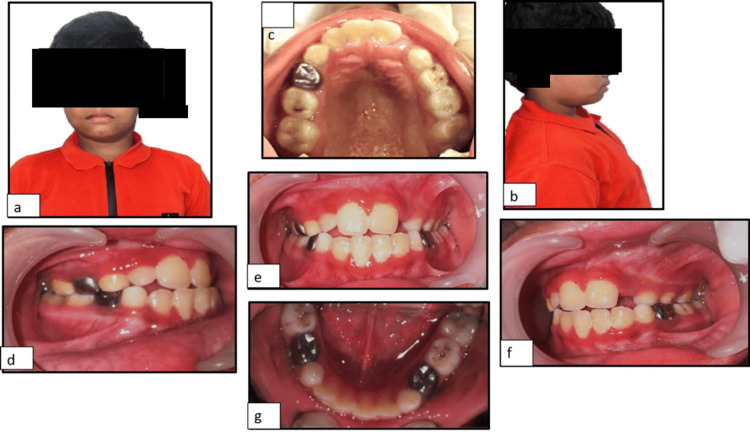
Intraoral pre-treatment photographs: (a) frontal view; (b) right lateral view; (c) maxillary occlusal view; (d) right buccal occlusion; (e) centric occlusion; (f) left buccal occlusion; (g) mandibular occlusal view.

Intraoral clinical examination (Figure 1) revealed a mixed dentition stage with a dental age corresponding to eight to nine years, with the maxillary left lateral incisor (22) in the process of active eruption. The maxilla appeared micrognathic about the mandible, contributing to a mild skeletal Class III malocclusion. An edge-to-edge anterior incisal relationship was observed, along with a posterior crossbite involving the maxillary left deciduous canines and molars, i.e., 63, 64. The patient had a history of dental treatment at the age of five years, which included stainless steel crowns on teeth 54, 74, and 84. Pit caries was noted on teeth 55, 64, 65, and 75, along with deep retentive fissures on the permanent first molars. Oral hygiene was inadequate, with a visible materia alba, indicating the need for improved preventive care and patient education. The patient presented with multiple functional, skeletal, and dental concerns. Functionally, there was evidence of deviated or abnormal swallowing patterns and a tongue thrust habit, which was a possible etiologic factor in the developing malocclusion.

The malocclusion and posterior crossbite warranted interceptive orthodontic intervention, possibly through orthopedic maxillary expansion, in conjunction with restorative and preventive dental management.

After assessing the functional, skeletal, and dental considerations based on the records (photographs, lateral cephalogram, and study models), a problem list was made. To address the chief concern, i.e., maxillary deficiency, a three-dimensional arch expansion was planned with a fixed appliance. The parent and the child were explained the treatment protocol, and the parent’s consent was obtained.

A three-dimensional expansion appliance was designed to achieve anterior and posterior expansion simultaneously to compensate for the maxillary constriction (Figure 2). It was a Hyrax-type RME with an acrylic bonded design. Such an appliance is used primarily to correct transverse maxillary deficiencies, such as posterior crossbites and narrow maxillary arches. The design included anterior wire extensions or acrylic coverage over anterior teeth, facilitating not only posterior expansion but also forward and lateral displacement of the anterior maxilla, which is crucial in patients presenting with edge-to-edge incisor relationships or mild skeletal Class III tendencies, as it aids in protracting the maxilla relative to the mandible. Posterior expansion addresses crossbite by widening the maxillary arch, particularly in the premolar and molar regions. This appliance is most effective in growing patients, particularly between the ages of 7 and 12 years, when the mid-palatal suture is still amenable to orthopedic modification. It was typically activated twice daily (achieving 0.25 mm separation per turn) for about two weeks, followed by a retention phase until the complete eruption of permanent canines. The overall treatment goal was to harmonize the skeletal discrepancy, relieve crossbite, and establish a more favorable Class I occlusion. 

**Figure 2:**
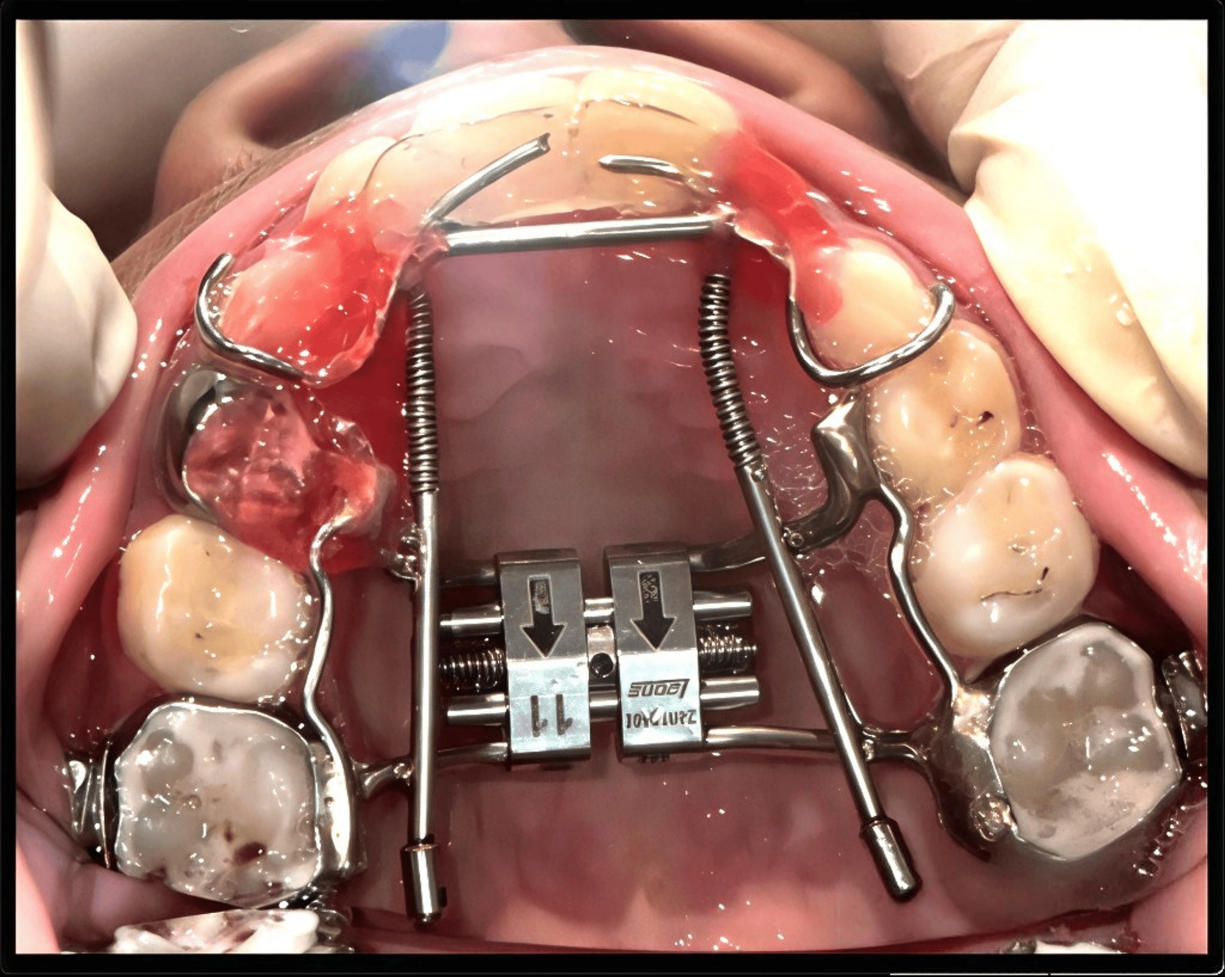
One week post-treatment: orthodontic relief wax applied.

Informed consent was obtained from the patient's parents before initiating the treatment. They were made aware of the nature and purpose of the rapid maxillary expansion, potential discomfort, necessary dietary restrictions, the importance of maintaining oral hygiene, and the commitment required throughout the process. The parents acknowledged understanding all aspects and willingly consented to proceed with the orthodontic intervention.

One week after appliance delivery, the patient reported wire impingement in the canine region. Orthodontic relief wax was prescribed to alleviate the discomfort (Figure 2). The complaint of discomfort with the anterior segment was persistent; hence, the canine stops were placed using resin-modified glass ionomer cement (Figure 3). After 30 days, the patient reported with a broken anterior segment of the expansion appliance (Figure 4). The posterior segment was retained for the next two weeks until the desired posterior expansion was obtained. Figure 5 shows intraoral status post-removal of the maxillary expansion appliance (after six weeks).

**Figure 3:**
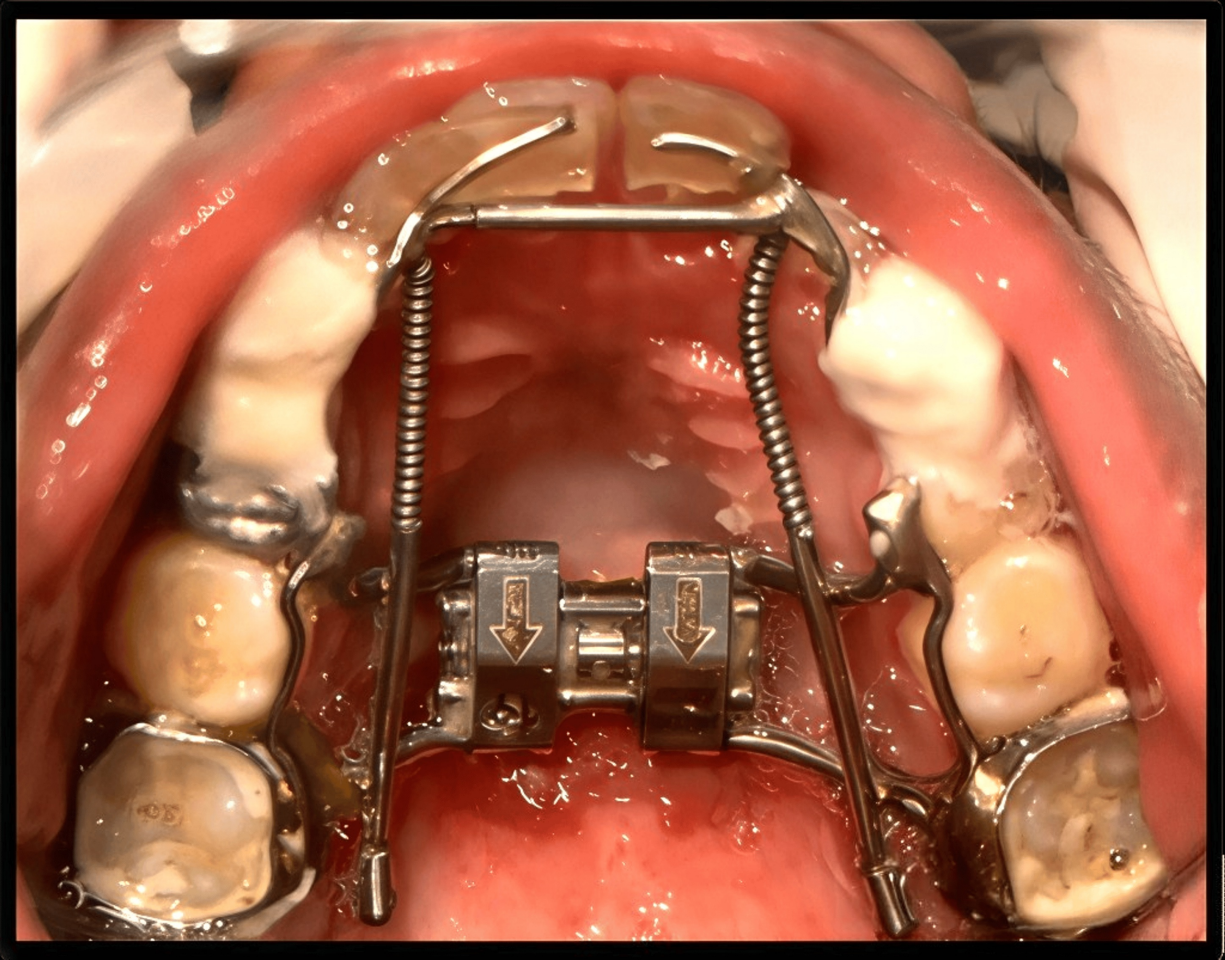
Three weeks after appliance placement: placement of canine stops using glass ionomer cement.

**Figure 4:**
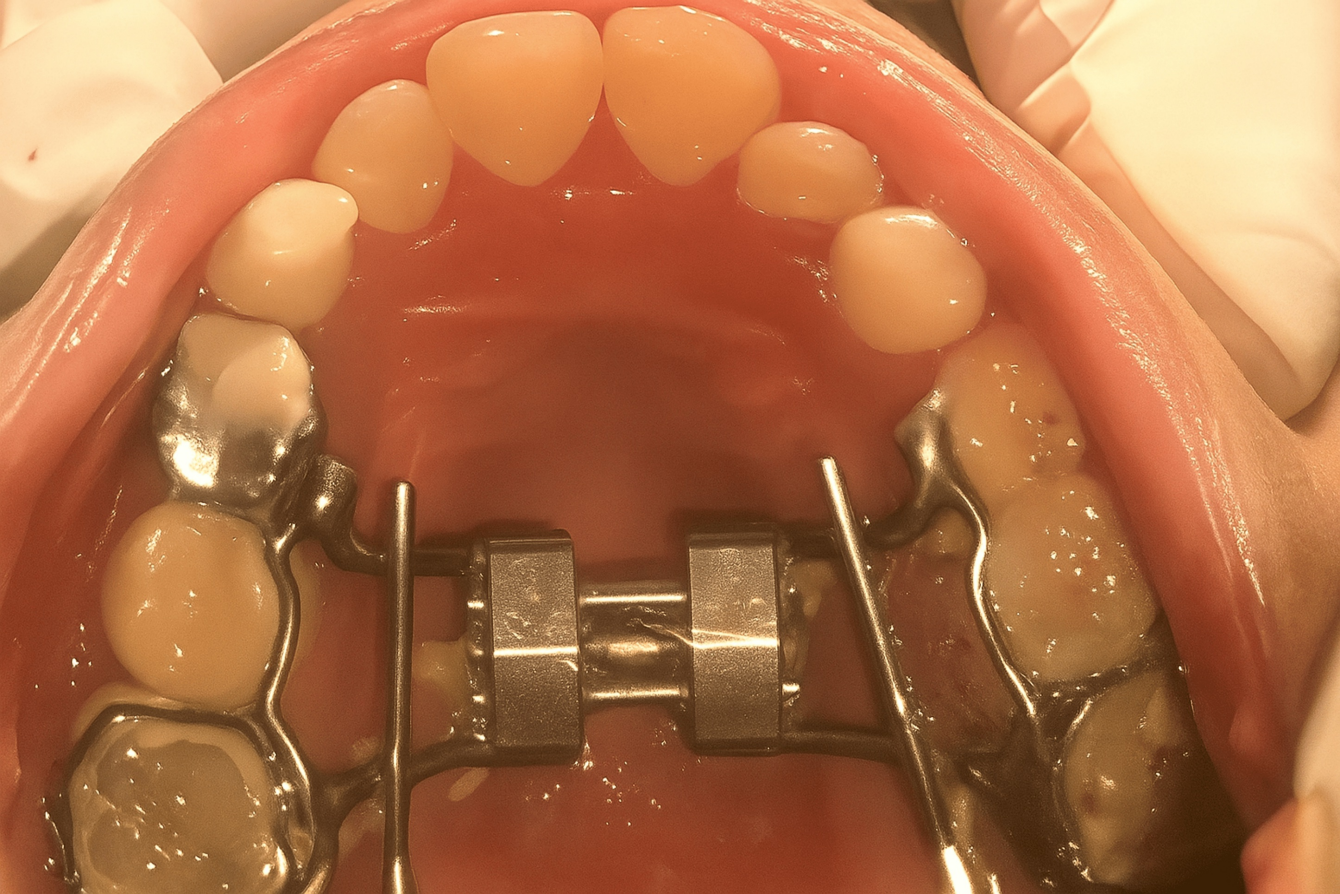
Four-week follow-up showing a retained posterior segment and a broken anterior segment of the expansion appliance.

**Figure 5:**
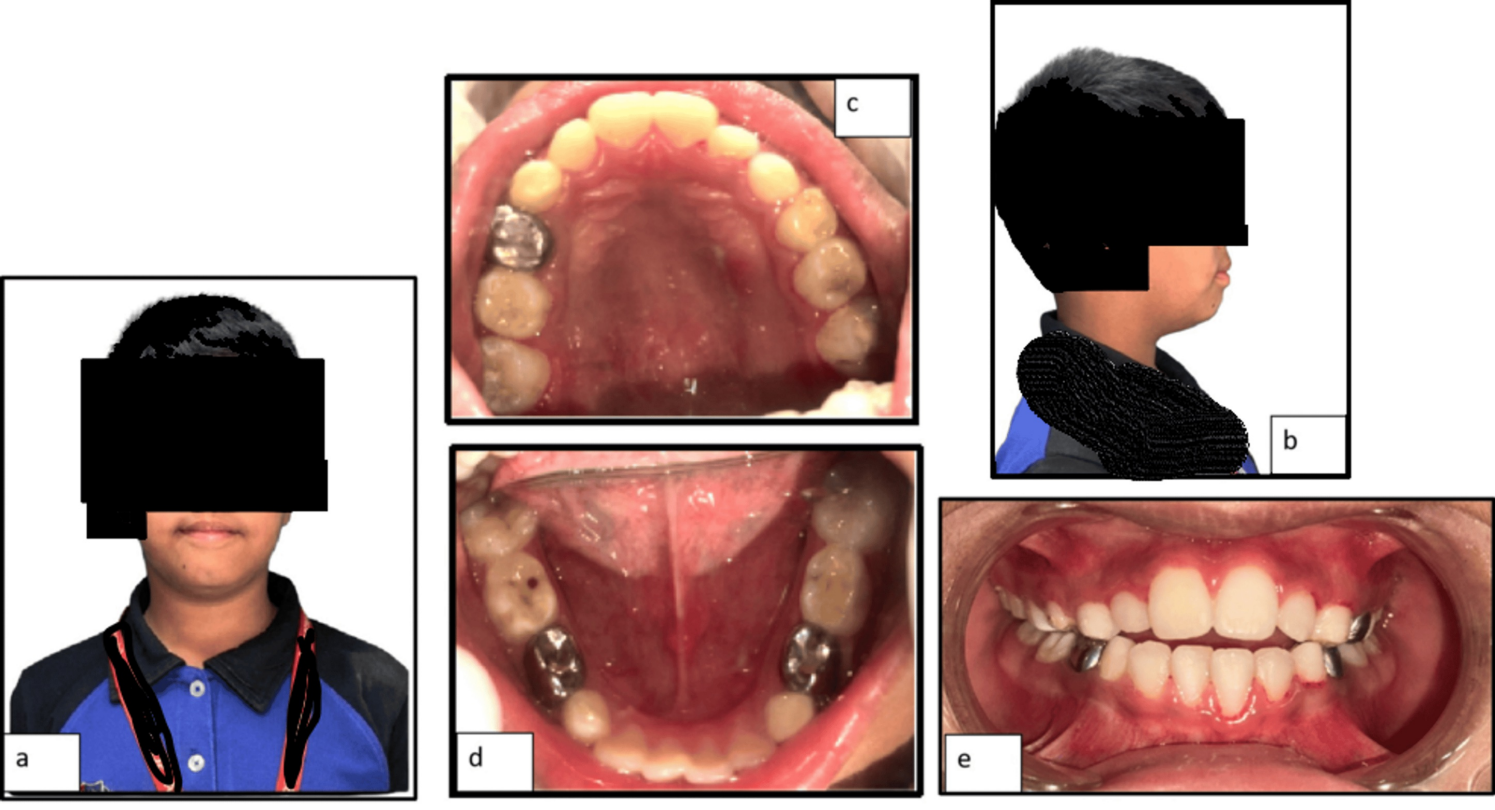
Intraoral photographs at six-week follow-up: (a) frontal view; (b) right lateral view; (c) maxillary occlusal view; (d) mandibular occlusal view; (e) centric occlusion.

After removal of the expansion appliance, a palatal crib appliance was delivered to the patient in order to control the tongue thrust habit, which led to an anterior open bite (Figure 6). Figure 7 shows the reduced anterior open bite after six weeks of palatal crib cementation.

**Figure 6:**
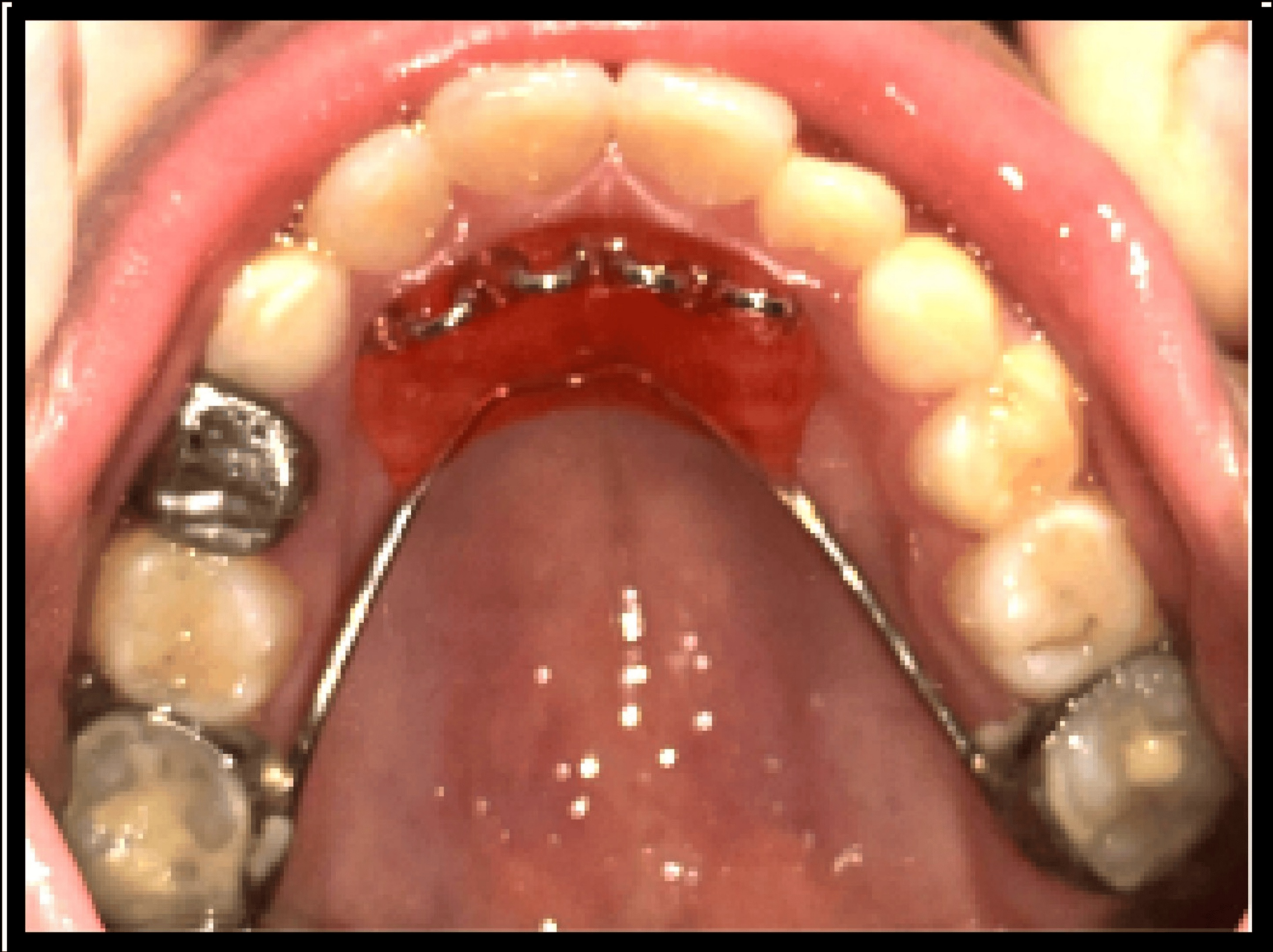
Palatal crib placed to address developing tongue thrust habit.

**Figure 7:**
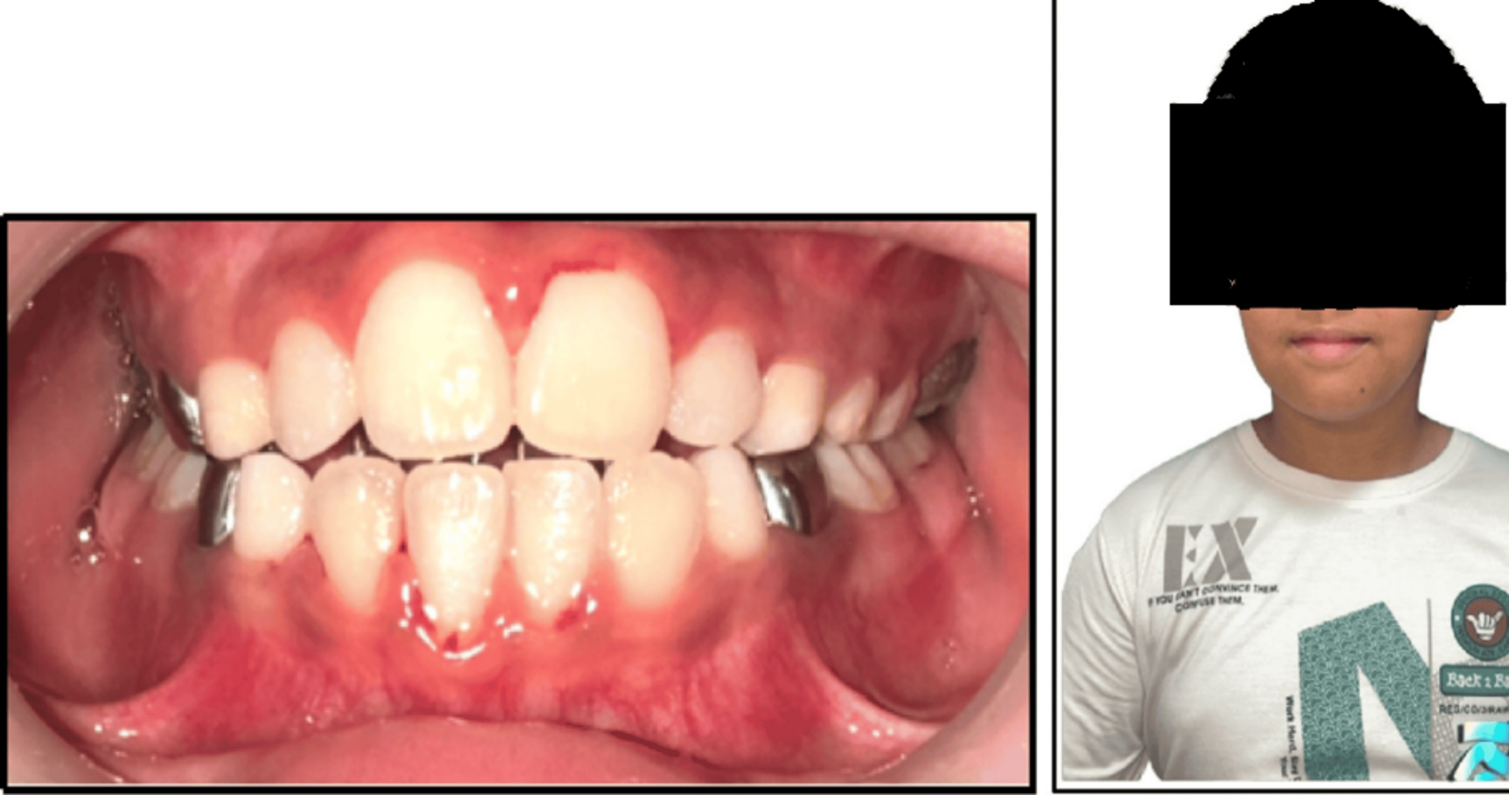
Follow-up at six weeks after palatal crib cementation: (a) centric occlusion; (b) frontal view.

The cephalometric comparison between pre-treatment and post-treatment values, as mentioned in Table 1, reveals significant skeletal and dentoalveolar changes, indicating successful orthopedic and orthodontic intervention (Figure 8).

**Table 1:** Comparison of cephalometric analysis: pre-treatment and post-treatment. PFH, posterior facial height; AFH, anterior facial height; SNA, sella-nasion-point A angle; SNB, sella-nasion-point B angle; ANB, A point-nasion-B point angle; SN-OP, sella-nasion to occlusal plane angle; SN-MP, sella-nasion to mandibular plane angle; U1 to FH, upper incisor to Frankfort horizontal angle; L1 to MP, lower incisor to mandibular plane angle; FMA, Frankfort mandibular angle; IMPA, incisor mandibular plane angle; FMIA, Frankfort mandibular incisor angle

	Parameter	Pre-treatment	Post-treatment	Remarks
Jarabak’s analysis	Jarabak’s ratio	66.0	71.28	Indicates a more horizontal growth pattern (>65%)
	PFH	68	72	More protrusive maxilla
	AFH	103	101	Slightly more protrusive mandible in Ceph 2
Steiner’s Analysis	SNA	79°	85°	Both show a Class I skeletal relationship
	SNB	82°	83°	Indicates maxillary occlusal plane inclination
	ANB	2°	2°	Slightly less vertical mandibular growth
	SN-OP	10°	12°	Slightly more open angle in Ceph 2
	SN-MP	33°	29°	Slightly more proclined upper incisors in Ceph 2
	Gonial Angle	130°	132°	Much more proclined lower incisors in Ceph 2
	U1 to FH	114°	116°	Slightly reduced → correlates with increased incisor proclination
	L1 to MP	79°	98°	Ceph1: High angle; ceph2: Low angle → flatter mandibular plane
	Overjet/Overbite	-	-	Slightly more proclined lower incisors in Ceph 2
	Interincisal Angle	137°	129°	Significantly reduced in Ceph 2 → more incisor proclination
Tweed’s Analysis	FMA	28°	22°	Decreased → More horizontal growth pattern post-treatment
	IMPA	95°	98°	Increased → Mandibular incisors are proclined post-treatment
	FMIA	58°	41°	Decreased → Indicates more protrusive lower incisors post-treatment

**Figure 8:**
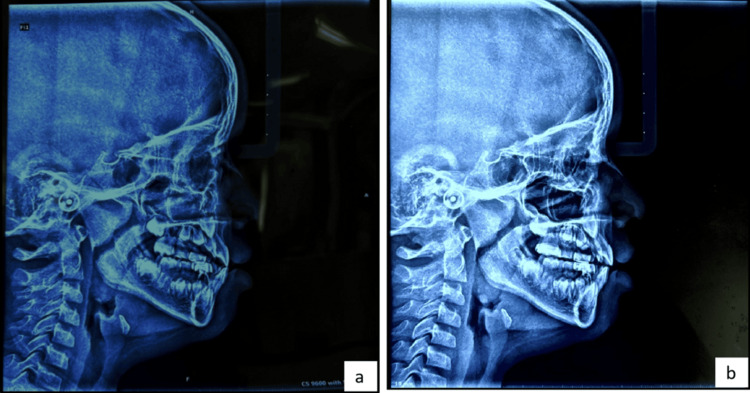
Lateral cephalogram: (a) pre-treatment; (b) post-treatment.

Growth pattern and facial proportions were assessed using Jarabak’s analysis. The increase in Jarabak’s ratio from 66.0 to 71.28 suggests a shift toward a more horizontal growth pattern, which is a favorable outcome in managing Class III tendencies. The increase in posterior facial height (PFH) alongside a slight reduction in anterior facial height (AFH) further supports this horizontal pattern, implying improved vertical control and better mandibular rotation.

Skeletal relationships and dentoalveolar changes were analyzed using Steiner’s analysis, which showed notable skeletal changes. The SNA angle increased from 79° to 85°, reflecting significant forward displacement or protraction of the maxilla, which aligns with the treatment goal of anterior maxillary advancement in a patient with maxillary retrognathism. The SNB angle also increased slightly (82°-83°), but the ANB remained constant at 2°, indicating maintenance of a skeletal Class I relationship while improving the maxillary position. The increase in SN-OP angle (10°-12°) indicates a slight steepening of the occlusal plane, while the SN-MP angle reduced from 33° to 29°, supporting a more favorable horizontal mandibular growth and a flattening of the mandibular plane angle.

Dental compensation is evident in the proclination of incisors. The U1 to FH angle increased from 114° to 116°, and the L1 to MP angle significantly increased from 79° to 98°, indicating pronounced lower incisor proclination post-treatment. This is further confirmed by the reduction in the interincisal angle to 129°, which typically correlates with more protrusive incisors and reduced overbite.

Tweed’s analysis reveals decreased FMA from 28° to 22°, again suggesting a more horizontal growth pattern and mandibular counter-clockwise rotation. The IMPA increased (95°-98°), and FMIA dropped significantly (58°-41°), showing increased incisor proclination, especially of the lower incisors, to compensate for the skeletal discrepancy.

Overall, the cephalometric changes demonstrate successful orthopedic correction of the maxillary constriction with improvement in both sagittal and transverse planes. The forward displacement of the maxilla, improved occlusal relationships, and controlled mandibular growth collectively indicate that the treatment approach was effective in addressing the underlying skeletal and dental issues associated with mild Class III malocclusion and maxillary deficiency.

## Discussion

Treating a developed Class III malocclusion is challenging due to its primarily skeletal nature, often involving maxillary deficiency, mandibular prognathism, or both. Once facial growth is complete, typically after adolescence, orthopedic interventions become ineffective, limiting options to dental camouflage or orthognathic surgery [11]. If diagnosis and intervention are delayed beyond the growth period, the malocclusion tends to worsen, making non-surgical correction more difficult [12].

Additionally, patients with Class III tendencies often show continued forward mandibular growth into late adolescence, increasing the risk of relapse [13]. Long-standing cases also present with functional shifts and dental compensations, such as retroclined lower incisors and proclined upper incisors, which complicate decompensation during pre-surgical orthodontics [14]. Esthetically, the concave facial profile associated with Class III malocclusion can negatively impact a patient’s self-esteem and quality of life, often necessitating comprehensive planning involving surgery for optimal results [15]. In adults with severe skeletal discrepancies, orthognathic surgery is usually the only effective treatment, which adds to the cost, invasiveness, and psychological burden of care [16].

Posterior crossbite affects 8-23% of children in the mixed dentition stage [1]. It may be unilateral or bilateral and is often associated with a narrow maxillary arch, leading to occlusal interferences and mandibular displacement. Early diagnosis through clinical and radiographic assessment, including occlusal examination and dental cast evaluation, is essential [17].

Intervention during the mixed dentition period is advocated to utilize the growth potential of the midpalatal suture before its increasing interdigitation with age [3,18]. The optimal timing for RME is during the prepubertal growth phase, as skeletal changes are more predictable, and relapse is less likely compared to post-pubertal expansion, which often produces more dental tipping than skeletal change [3]. RME applies heavy, intermittent forces to open the midpalatal suture, resulting in skeletal widening of the maxillary arch. The Hyrax and Haas appliances are commonly used, with activation protocols ranging from 0.25 mm to 0.5 mm per day [19,20]. Studies have shown that RME not only corrects the crossbite but also improves nasal airway volume and reduces mouth breathing [21,22]. While RME is largely safe in growing children, potential side effects include gingival recession, root resorption, and tipping of anchor teeth. These risks are minimized with correct appliance design, controlled activation, and adequate retention.

In a child aged 9 years, the suture is still relatively patent, allowing successful skeletal expansion with minimal adverse effects. RME at this age results in increased intermolar and intercanine widths, improves arch coordination, and often resolves functional shifts [19].

While the orthodontic interventions in this case yielded favorable outcomes, several skeletal and dental limitations were identified that would have impacted the treatment planning and prognosis. Skeletally, the primary limitation was the underlying maxillary retrognathism, which required timely orthopedic correction. The success of maxillary expansion is closely linked to growth potential, as its effectiveness diminishes as skeletal maturity progresses. The reduced vertical lower facial height and the mildly retruded chin also presented challenges in achieving ideal vertical proportions and profile balance.

Dentally, significantly lower incisor proclination served as a compensatory mechanism for the skeletal discrepancy, limiting the extent of further dental movement. The presence of an edge-to-edge bite, developing dentition, and suboptimal oral hygiene further complicated treatment. Appliance-related issues, including anterior segment breakage and patient discomfort, highlighted the need for durable appliance design and patient cooperation. Appliance-related factors, including breakage of the anterior segment of the appliance and patient discomfort, introduced challenges in maintaining consistent activation and retention, underscoring the importance of appliance design durability and patient compliance.

Moreover, the case was managed during the mixed dentition phase, with several teeth still erupting. This limited the feasibility of comprehensive fixed orthodontic therapy at this stage and necessitated a phased approach. Inadequate oral hygiene and the presence of active caries risk posed additional challenges, emphasizing the need for integrated preventive care alongside interceptive orthodontics.

Despite these limitations, timely intervention with a customized 3D expansion appliance, coupled with habit control measures and functional evaluation, enabled substantial improvement in skeletal relationships, occlusion, and facial esthetics. However, in order to ensure the stability and completeness of correction, long-term follow-up till the age of 12 years and possibly a second phase of treatment in permanent dentition would be essential. Parents' perspective toward the treatment offered was as stated: "Although the treatment journey involved some initial discomfort and required dietary adjustments and extra care for oral hygiene, the positive changes and outcome made it all worthwhile."

## Conclusions

The effective application of a 3D Hyrax-type RME with anterior and posterior expansion capabilities in a growing patient presenting with maxillary constriction, edge-to-edge incisor relationship, and mild skeletal Class III malocclusion is evident in this case report. The bonded acrylic design, with anterior extensions, facilitated both transverse and sagittal correction, addressing the underlying skeletal discrepancy. Despite a minor complication involving breakage of the anterior segment at the four-week follow-up, the posterior component continued to function effectively, allowing the targeted posterior expansion to be completed successfully. The subsequent use of a palatal crib appliance rendered stabilization of the results, control of tongue thrust, and led to better incisor relation. This case showcases the importance of early intervention, appliance design customization, and close monitoring to manage developing skeletal imbalances and achieve favorable long-term occlusal and orthopedic outcomes.
